# Radiative MHD Nanofluid Flow over a Moving Thin Needle with Entropy Generation in a Porous Medium with Dust Particles and Hall Current

**DOI:** 10.3390/e22030354

**Published:** 2020-03-18

**Authors:** Iskander Tlili, Muhammad Ramzan, Seifedine Kadry, Hyun-Woo Kim, Yunyoung Nam

**Affiliations:** 1 Department for Management of Science and Technology Development, Ton Duc Thang University, Ho Chi Minh City 758307, Vietnam; iskander.tlili@tdtu.edu.vn; 2 Faculty of Applied Sciences, Ton Duc Thang University, Ho Chi Minh City 758307, Vietnam; 3 Department of Computer Science, Bahria University, Islamabad Campus, Islamabad 44000, Pakistan; 4 Department of Mathematics and Computer Science, Faculty of Science, Beirut Arab University, Beirut 115020, Lebanon; s.kadry@bau.edu.lb; 5 Department of ICT Convergence Rehabilitation Engineering, Soonchunhyang University, Asan 31538, Korea; lovekhw@gmail.com; 6 Department of Computer Science and Engineering, Soonchunhyang University, Asan 31538, Korea

**Keywords:** entropy generation, nonlinear thermal radiation, energy conservation, magnetohydrodynamic, nanofluid, thin needle

## Abstract

This paper investigated the behavior of the two-dimensional magnetohydrodynamics (MHD) nanofluid flow of water-based suspended carbon nanotubes (CNTs) with entropy generation and nonlinear thermal radiation in a Darcy–Forchheimer porous medium over a moving horizontal thin needle. The study also incorporated the effects of Hall current, magnetohydrodynamics, and viscous dissipation on dust particles. The said flow model was described using high order partial differential equations. An appropriate set of transformations was used to reduce the order of these equations. The reduced system was then solved by using a MATLAB tool bvp4c. The results obtained were compared with the existing literature, and excellent harmony was achieved in this regard. The results were presented using graphs and tables with coherent discussion. It was comprehended that Hall current parameter intensified the velocity profiles for both CNTs. Furthermore, it was perceived that the Bejan number boosted for higher values of Darcy–Forchheimer number.

## 1. Introduction 

The novel idea to reduce entropy generation in heat transfer convective processes was floated by Bejan [1]. In thermodynamic systems, this notion is employed to enhance the efficiency of thermal engineering gadgets [2]. Indeed, entropy generation is used to gauge the molecular chaos or disorder in a thermodynamic system. Thermodynamics’ second law states that higher molecular disorder is inversely proportional to the quality of energy reduction. It has been examined that energy dissipation and heat transfer owing to differences in temperatures are the key factors for entropy generation. That is why special attention is given for the enhancement of heat transfer in varied engineering applications. The average internal heat loss because of entropy generation in a titanium dioxide (TiO2) suspended in a water-based nanofluid Poiseuille flow with the impact of mixed convection and thermal radiation in a wavy channel is discussed by Zeeshan et al. [3]. The 2nd law of thermodynamics is betrothed for the entropy generation model erection. It is reported that the pressure gradient is the key factor for a rise in the average energy loss. Further, it is noted that the entropy generation for the radiation parameter is more near walls in comparison to the middle of the channel. The study of entropy generation in the nanofluid thin-film flow, containing a suspension of both types of carbon nanotubes (CNTs) with Cattaneo-Christov heat flux, magnetohydrodynamics, and variable source/sink, is studied by Lu et al. [4]. Numerical simulations with the erected mathematical model are found by the bvp4c function of MATLAB software (University of New Mexico, New Mexico). It is witnessed that entropy generation is larger for higher estimates of the magnetic parameter for a thin film flow. The entropy generation analysis during the heat transfer process in the flow of Ferrofluid with low oscillating magnetic field past a stretched rotating disk is deliberated by Hassan et al. [5]. The analytical solution of the problem is attained via Mathematica-based bvp 2.0 based on the homotopy analysis method. It is comprehended that total entropy is boosted with the dispersion of nanoparticles. Further, it is noted that the irreversibility of the fluid flow is enhanced by the strong magnetic impact. Some recent literature about entropy generation may be found in references [6,7,8,9,10]. 

CNTs are cylindrical type carbon allotropes. These were first exposed in the form of multi-walled carbon nanotubes (MWCNTs) by Iijima [11] in 1991. This was followed by another study by Bethune et al. [12] who introduced the idea of single-walled carbon nanotubes (SWCNTs) in 1993. In today’s era, a good number of applications involving CNTs may be found, like in health care, energy, electronics, etc. [13,14,15]. It is now a well-established theory that snags with the materials possessing low thermal conductivity are removed with the introduction of nanofluids. Nanofluids holds nanoparticles with a size of <100 nm. These nanoparticles are made up of copper, metal oxides, alumina, nanomaterials, nitrides, and carbides [16]. The concept of nanofluids was the first time floated by Choi and Eastman [17]. A substantial number of studies have been carried out since its inception [18,19,20]. Recently, Sheikholeslami and Shehzad [21] numerically examined the flow of nanofluid comprising Fe_3_O_4_-H_2_O solution in a permeable cavity under the influence of a variable magnetic field using Control Volume Finite Element Method (CVFEM). They observed the highest heat transfer rate in the case of the platelet-shaped nanoparticles. It was further witnessed by them that the velocity of the nanofluid was on the decline once the strong magnetic field was applied. Entropy optimization for the flow of Carreau nanofluid flow with cubic auto-catalysis chemical reaction was studied by Khan et al. [22] analytically. They noticed that the sturdier magnetic field boosted the entropy generation. Sheikholeslami [23] found a numerical solution of nanofluid flow under the influence of the magnetic field in a permeable medium via the CVFEM scheme. He analyzed the influences of entropy and exergy on the presented model and reported that entropy loss enhanced in attendance of stronger magnetic field. Khan et al. [24] examined the numerical solution of 3D cross nanofluid with activation energy and binary chemical reaction with zero mass flux and convective boundary conditions. They noticed that higher estimates of activation energy boosted the concentration of the cross nanofluid. Hosseini and Sheikholeslami [25] analyzed the thermal competence of a convective nanofluid flow with entropy generation inside a microchannel under the influence of the magnetic field. Between two phases, non-equilibrium condition for a permeable media is engaged. They noticed that the entropy generation enhanced with an increase in fluid friction irreversibility. Some recent studies have also highlighted the concept of carbon nanotubes [26,27,28,29,30,31,32,33] and many therein. 

Abundant applications focusing on Cattaneo-Christov heat flux amalgamated with thermal radiation may be found in missiles, air crafts, nuclear power plants, space vehicles’ propulsion gadgets, etc. Keeping in view these interesting applications, scientists and researchers [34,35,36,37,38,39,40] are motivated to look for fluid behavior in attendance of thermal radiation and Cattaneo-Christov heat flux. 

Motivated from the above literature, our objective was to find the water-based CNTs dusty nanofluid flow over a moving thin needle. The analysis was performed in the presence of Hall current and nonlinear thermal radiation in a Darcy–Forchheimer porous media. The thermal efficacy of the system was analyzed by employing entropy analysis. A numerical solution of the envisaged inimitable mathematical model was found. To our information, no such study has been conducted so far in the literature. This model was unique in its category. Endorsement of the outcomes of the existing study was done by comparing with a published article in limiting case. Graphical sketches and tables were also part of this study.

## 2. Mathematical Modeling

**Figure 1:**
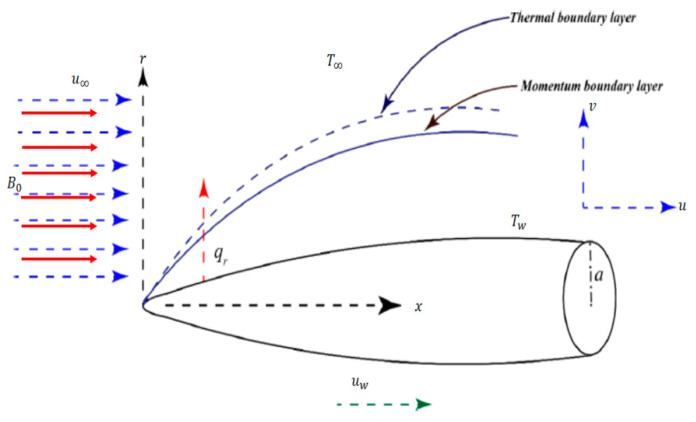
The physical design of the flow problem.

Let us assume an H_2_O-CNTs-based nanofluid flow with Hall current over a moving slender needle having speed $u_{w}$ and radius “*a*” (Figure 1). The speed of fluid far away from the surface is taken as $u_{\infty }.$ The cylindrical coordinates *(x, r)* are taken in such a way that x− is along the axis of the needle and r− normal to the axis. The flow containing dust particles is generated in a non-Darcy absorbent media. The associated impacts affecting the flow in the heat equation are viscous dissipation and nonlinear thermal radiation. Furthermore, $T_{w}$ and $T_{\infty }$ are the constant temperatures at the wall and far off from the wall with $T_{\infty }>T_{w}.$ A magnetic field with magnetic strength $B_{0}$ is applied with the low Reynold number assumption [41], which eventually results in the induced magnetic field to be neglected. Two types of the equations, i.e., fluid phase and particle phase, comprising the envisioned mathematical model fulfilling laws of conservation are also laid down as given below:

The above flow theory gives rise to the following boundary layer equations [41,42,43,44,45]:

Continuity equation:
  Fluid phase
  (1) $$\frac{\partial (ru)}{\partial x}+\frac{\partial (rv)}{\partial r}=0,$$
Momentum equation:
  Fluid phase
  (2) $$\begin{array}{c} \left(1-\phi_{d}\right)\left(u\frac{\partial u}{\partial x}+v\frac{\partial u}{\partial r}\right)=\left(1-\phi_{d}\right)\left(\frac{\mu_{nf}}{\rho_{nf}}\frac{1}{r}\frac{\partial }{\partial r}\left(r\frac{\partial u}{\partial r}\right)\right)-\frac{\nu_{nf}}{k^{*}}u\\ -\frac{C_{b}}{x\sqrt{k^{*}}}u^{2}+\frac{KN}{\rho_{nf}}\left(u_{p}-u\right)-\frac{\sigma B_{0}{}^{2}}{\rho_{nf}\left(1+m^{2}\right)}u, \end{array}$$
Continuity equation:
  Particle phase
  (3) $$\frac{\partial \left(ru_{p}\right)}{\partial x}+\frac{\partial \left(rv_{p}\right)}{\partial r}=0,$$
Momentum equation:
  Particle phase
  (4) $$u_{p}\frac{\partial u_{p}}{\partial x}+v_{p}\frac{\partial u_{p}}{\partial r}=\frac{K}{m_{d}}\left(u-u_{p}\right),$$
Energy equation:
  Fluid phase
  (5) $$\begin{array}{c} u\frac{\partial T}{\partial x}+v\frac{\partial T}{\partial r}=\frac{k_{nf}}{{\left(\rho C_{p}\right)}_{nf}}\frac{1}{r}\frac{\partial }{\partial r}\left(r\frac{\partial T}{\partial r}\right)-\frac{1}{{\left(\rho C_{p}\right)}_{nf}}\frac{\partial q_{r}}{\partial r}+\frac{\mu_{nf}}{{\left(\rho C_{p}\right)}_{nf}}{\left(\frac{\partial u}{\partial r}\right)}^{2}\\ +\frac{\mu_{nf}}{k^{*}{\left(\rho C_{p}\right)}_{nf}}u^{2}+\frac{N_{1}}{\tau_{\upsilon }{\left(\rho C_{p}\right)}_{nf}}{\left(u_{p}-u\right)}^{2}+\frac{N_{1}{\left(C_{p}\right)}_{nf}}{\tau_{T}{\left(\rho C_{p}\right)}_{nf}}\left(T_{p}-T\right)\\ +\frac{\sigma B_{0}{}^{2}}{{\left(\rho C_{p}\right)}_{nf}\left(1+m^{2}\right)}u^{2}, \end{array}$$
Energy equation:
  Particle phase
  (6) $$N_{1}c_{m}\left(u_{p}\frac{\partial T_{p}}{\partial x}+v_{p}\frac{\partial T_{p}}{\partial r}\right)=\frac{N_{1}{\left(C_{p}\right)}_{nf}}{\tau_{T}}\left(T_{p}-T\right),$$

with the boundary conditions

(7) $$u\left(x,r\right)=u_{w},\;\;v\left(x,r\right)=0,u_{p}\left(x,r\right)=u_{w},\;\;v_{p}\left(x,r\right)=0,T\left(x,r\right)=T_{w},\;\;\;at\;\;r=R\left(x\right),$$

(8) $$u\left(x,r\right)\;\to \;u_{\infty },\;\;\;\;T\left(x,r\right)\to \;T_{\infty },\;T_{p}\left(x,r\right)\to T_{\infty }\;\;as\;r\to \infty .$$

The heat flux in simplified form after considering Rosseland approximation is:

(9) $$q_{r}=-\frac{4\sigma_{SB}}{3a_{r}}\frac{\partial T^{4}}{\partial r},$$

where $a_{r}$ is the mean absorption coefficient, and $\sigma_{SB}$ is the Stephen–Boltzman constant. For a planer boundary layer flow, the above equation can be written as:

(10) $$q_{r}=-\frac{16\sigma_{SB}}{3a_{r}}T^{3}\frac{\partial T}{\partial r}.$$

The mathematical model proposed by Xue [46] for the CNTs is given in Table 1. The thermo-physical traits of the CNTs of both types and H_2_O are appended in Table 2.

## 3. Similarity Transformation

The similarity variables are introduced as follows,

(11) $$\begin{array}{c} u=2Ug^{\prime }\left(\xi \right),\;\;\;\;\;u_{p}=2UG^{\prime }\left(\xi \right),\;\;\;\;v=\frac{Ur}{x}g^{\prime }\left(\xi \right)-\frac{\upsilon_{bf}}{r}g\left(\xi \right),\\ v_{p}=\frac{Ur}{x}G^{\prime }\left(\xi \right)-\frac{\upsilon_{bf}}{r}G\left(\xi \right),\;\xi =\frac{Ur^{2}}{\upsilon_{bf}x},\;\;\;\;\;\theta \left(\xi \right)=\frac{T-T_{\infty }}{T_{w}-T_{\infty }},\;\theta_{p}\left(\xi \right)=\frac{T_{p}-T_{\infty }}{T_{w}-T_{\infty }}. \end{array}$$

The resulting non-dimensional Ordinary differential equations (ODEs) system after referring to similarity variables:

Momentum equation:
  Fluid phase
  (12) $$\begin{array}{l} \left(1-\phi_{d}\right){\left(1-\phi \right)}^{2.5}\left(1-\phi +\phi \frac{\rho_{CNT}}{\rho_{bf}}\right)gg^{\prime \prime }+2\left(g^{\prime \prime }+\xi g^{\prime \prime \prime }\right)-\lambda g^{\prime }\\ \;-{\left(1-\phi \right)}^{2.5}\left(1-\phi +\phi \frac{\rho_{CNT}}{\rho_{bf}}\right)F_{r}{g^{\prime }}^{2}+{\left(1-\phi \right)}^{2.5}\alpha \beta \left(G^{\prime }-g^{\prime }\right)\\ \;-{\left(1-\phi \right)}^{2.5}\frac{M}{1+m^{2}}g\prime =0, \end{array}$$
Momentum equation:
  Particle phase
  (13) $$G^{\prime \prime }G+\beta \left(g^{\prime }-G\prime \right)=0,$$
Energy equation:
  Fluid phase
  (14) $$\begin{array}{l} \frac{k_{nf}}{k_{bf}}\left(\xi \theta^{\prime \prime }+\theta \prime \right)+0.5Pr\left(1-\phi +\phi \frac{{\left(\rho C_{p}\right)}_{CNT}}{{\left(\rho C_{p}\right)}_{bf}}\right)g\theta^{\prime }\\ \;+\frac{4}{3N_{r}}{\left(1+\left(\theta_{r}-1\right)\theta \right)}^{2}\{3\xi \left(\theta_{r}-1\right)\theta \prime^{2}\\ \;+\left(1+\left(\theta_{r}-1\right)\theta \right)\left(0.5\theta^{\prime }+\xi \theta \prime \prime \right)\}+\frac{4EcPr}{{\left(1-\phi \right)}^{2.5}}\xi g^{\prime \prime 2}\\ \;+2\alpha \beta EcPr{\left(G^{\prime }-g^{\prime }\right)}^{2}+2EcPr\left(\frac{\lambda }{{\left(1-\phi \right)}^{2.5}}+\frac{M}{1+m^{2}}\right)g\prime^{2}\\ \;+0.5\alpha \beta_{T}Pr\left(1-\phi +\phi \frac{{\left(C_{p}\right)}_{CNT}}{{\left(C_{p}\right)}_{bf}}\right)\left(\theta_{p}-\theta \right)=0, \end{array}$$
Energy equation:
  Particle phase
  (15) $$G\theta_{p}^{\prime }-\gamma \beta_{T}\left(1-\phi +\phi \frac{{\left(C_{p}\right)}_{CNT}}{{\left(C_{p}\right)}_{bf}}\right)\left(\theta_{p}-\theta \right)=0,$$
  associated with boundary conditions
  (16) $$\begin{array}{c} g\left(a\right)=\frac{a}{2}\varepsilon ,\;\;g^{\prime }\left(a\right)=\frac{\varepsilon }{2},\;\;G\left(a\right)=\frac{a}{2}\varepsilon ,\;\;G^{\prime }\left(a\right)=\frac{\varepsilon }{2},\;\;\;\;\;\;\;\;\;\;\;\theta \left(a\right)=1,\\ g^{\prime }\left(\infty \right)\to \frac{1-\varepsilon }{2},\;\;\theta \left(\infty \right)\to 0,\;\;\theta_{p}\left(\infty \right)\;\to \;0. \end{array}$$

Here, prime represents derivative with respect to ξ. The dimensionless physical parameters are defined as follows:

(17) $$\begin{array}{c} \lambda =\frac{\upsilon_{bf}}{2Uk^{*}}\;,\;\;\;F_{r}=\frac{C_{b}}{\sqrt{k^{*}}}\;,\;\;\;\alpha =\frac{Nm_{d}}{\rho_{bf}}\;,\;\;\;\beta =\frac{K}{2Um_{d}}\;,\;M=\frac{\sigma B_{0}{}^{2}}{2U\rho_{bf}}\;,\;\;\;\\ Pr=\frac{\upsilon_{bf}}{k_{bf}}{\left(\rho C_{p}\right)}_{bf}\;,\;\;\;N_{r}=\frac{a_{r}k_{bf}}{4\sigma_{SB}T_{\infty }{}^{3}}\;,\;\;\;\theta_{r}=\frac{T_{w}}{T_{\infty }}\;,\;Ec=\frac{U^{2}}{\left(T_{w}-T_{\infty }\right){\left(C_{p}\right)}_{bf}}\;,\;\;\\ \beta_{T}=\frac{1}{2U\tau_{T}}\;,\;\gamma =\frac{{\left(C_{p}\right)}_{bf}}{c_{m}}. \end{array}$$

## 4. Nusselt Number and Skin Friction Coefficient

The skin friction coefficients $C_{fx}$ and the local Nusselt number $Nu_{x}$ in dimensional form are given by:

(18) $$C_{fx}=\frac{2\tau_{w}}{\rho_{bf}U^{2}},\;\;Nu_{x}=\frac{xq_{w}}{k_{bf}\left(T_{w}-T_{\infty }\right)},$$

where $\tau_{w}$ and $q_{w}$ are defined as below:

(19) $$\tau_{w}={\left[\mu_{nf}\frac{\partial u}{\partial r}\right]}_{r=a},\;\;q_{w}=-k_{nf}{\left(\frac{\partial T}{\partial r}\right)}_{r=a}+{\left(q_{r}\right)}_{r=a}.$$

By using Equations (11), (18) and (19) we get

(20) $$\sqrt{Re_{x}}C_{fx}=\frac{8a^{1/2}g^{\prime \prime }(a)}{{\left(1-\phi \right)}^{2.5}},$$

(21) $$\frac{Nu_{x}}{\sqrt{Re_{x}}}=-2a^{\frac{1}{2}}\left(\frac{k_{nf}}{k_{bf}}\right)\left(1+\frac{4}{3N_{r}}\theta_{r}{}^{3}\right)\theta^{\prime \left(a\right)}.$$

with

(22) $$Re_{x}=\frac{Ux}{\upsilon_{bf}}.$$

## 5. Entropy Generation 

Entropy generation analysis is much important to study the thermal energy irreversibility of a particular system.

(23) $${\dot{S}}^{\prime \prime \prime }{}_{GEN}=\underbrace{\begin{array}{c} \left[\begin{array}{c} \frac{k_{nf}}{T^{2}}{\left(\frac{\partial T}{\partial r}\right)}^{2}+\frac{k_{nf}}{T^{2}}\left\{\frac{16\sigma_{SB}}{3a_{r}k_{bf}}T^{3}{\left(\frac{\partial T}{\partial r}\right)}^{2}\right\}\\ Entropy\;due\;to\;heat\;transfer \end{array}\right]\\ +\underbrace{\left[\begin{array}{c} \frac{\mu_{nf}}{Tk^{*}}u^{2}+\frac{\mu_{nf}}{T}{\left(\frac{\partial T}{\partial r}\right)}^{2}\\ Energy\;due\;to\;fluid\;friction \end{array}\right]} \end{array}}+\underbrace{\left[\begin{array}{c} \frac{\sigma {B_{0}}^{2}}{T(1+m^{2})}u^{2}\\ Energy\;due\;to\;\mathrm{diffusion} \end{array}\right]},$$

Equation (23), after employing Equation (11), in dimensionless form is

(24) $$\begin{array}{l} N_{S}=\frac{k_{nf}}{k_{bf}}\xi {\left(\theta_{r}-1\right)}^{2}\theta^{\prime 2}(\frac{1}{{\left(1+\left(\theta_{r}-1\right)\theta \right)}^{2}}+\frac{4}{3N_{r}}\left(1+\left(\theta_{r}-1\right)\theta \right))\\ \;+\frac{4EcPr\left(\theta_{r}-1\right)}{{\left(1-\phi \right)}^{2.5}\left(1+\left(\theta_{r}-1\right)\theta \right)}\xi g{\prime \prime }^{2}+\frac{2EcPr\left(\theta_{r}-1\right)}{\left(1+\left(\theta_{r}-1\right)\theta \right)}\left(\frac{\lambda }{{\left(1-\phi \right)}^{2.5}}+\frac{M}{1+m^{2}}\right)g\prime^{2}. \end{array}$$

Here, the characteristic entropy generation is given by:

(25) $${\left({\dot{S}}^{\prime \prime \prime }{}_{GEN}\right)}_{0}=\frac{4k_{bf}U}{\upsilon_{bf}x},$$

In non-dimensional form, heat transfer irreversibility is given by:

(26) $$N_{HT}=\frac{k_{nf}}{k_{bf}}\xi {\left(\theta_{r}-1\right)}^{2}\theta \prime^{2}\left(\frac{1}{{\left(1+\left(\theta_{r}-1\right)\theta \right)}^{2}}+\frac{4}{3N_{r}}\left(1+\left(\theta_{r}-1\right)\theta \right)\right).$$

The fluid friction irreversibility is defined by:

(27) $$N_{FF}=\frac{4EcPr\left(\theta_{r}-1\right)}{{\left(1-\phi \right)}^{2.5}\left(1+\left(\theta_{r}-1\right)\theta \right)}\xi g^{\prime \prime 2},$$

and the porous medium and magnetic field irreversibility are represented by:

(28) $$N_{PMF}\frac{2EcPr\left(\theta_{r}-1\right)}{\left(1+\left(\theta_{r}-1\right)\theta \right)}\left(\frac{\lambda }{{\left(1-\phi \right)}^{2.5}}+\frac{M}{1+m^{2}}\right)g\prime^{2},$$

The Bejan number *Be* in dimensional form is defined as:

(29) $$Be=\frac{\frac{k_{nf}}{T^{2}}{\left(\frac{\partial T}{\partial r}\right)}^{2}+\frac{k_{nf}}{T^{2}}\left\{\frac{16\sigma_{SB}}{3a_{r}k_{bf}}T^{3}{\left(\frac{\partial T}{\partial r}\right)}^{2}\right\}}{\frac{k_{nf}}{T^{2}}{\left(\frac{\partial T}{\partial r}\right)}^{2}+\frac{k_{nf}}{T^{2}}\left\{\frac{16\sigma_{SB}}{3a_{r}k_{bf}}T^{3}{\left(\frac{\partial T}{\partial r}\right)}^{2}\right\}+\frac{\mu_{nf}}{Tk^{*}}u^{2}+\frac{\mu_{nf}}{T}{\left(\frac{\partial T}{\partial r}\right)}^{2}+\frac{\sigma B_{0}{}^{2}}{T\left(1+m^{2}\right)}u^{2}}.$$

In dimensionless form, Be after consulting (11) is:

(30) Be=(ξ(θr−1)2θ′2(3Nr+4(1+(θr−1)θ)3))(ξ(θr−1)2θ′2(3Nr+4(1+(θr−1)θ)3)+12NrEcPr(θr−1)(knfkbf)(1−ϕ)2.5ξ(1+(θr−1)θ)g″2+6NrEcPr(θr−1)(knfkbf)(1+(θr−1)θ)(λ(1−ϕ)2.5+M1+m2)),

## 6. Numerical Scheme

The system with high nonlinearity comprising Equations (12)–(15) with the support of Equation (16) is numerically solved by bvp4c MATLAB function. The following code transforms the given model into the 1st order system of ODEs.

(31) $$\begin{array}{l} g=y_{1},\\ g\prime =y_{2},\\ g\prime \prime =y_{3},\\ g\prime \prime \prime =yy_{1},\\ yy_{1}=\frac{1}{2\xi }\left[\begin{array}{l} \lambda y_{2}-2y_{3}+{\left(1-\varphi \right)}^{2.5}(1-\varphi +\varphi \frac{\rho_{CNT}}{\rho_{bf}})F_{r}y_{2}^{2}\\ -{\left(1-\varphi \right)}^{2.5}\alpha \beta (y_{5}-y_{2})-(1-\varphi_{d}){\left(1-\varphi \right)}^{2.5}(1-\varphi +\varphi \frac{\rho_{CNT}}{\rho_{bf}})y_{1}y_{3}\\ +{\left(1-\varphi \right)}^{2.5}\frac{M}{1+m^{2}y_{2}} \end{array}\right], \end{array}$$

(32) $$\begin{array}{l} \theta =y_{6},\\ \theta \prime =y_{7},\\ \theta \prime \prime =yy_{3}, \end{array}$$

(33) $$yy_{3}=\frac{1}{\frac{k_{nf}}{k_{bf}}\xi +\frac{4}{3Nr}{\left(1+(\theta_{r}-1)\theta \right)}^{3}}\left[\begin{array}{l} \frac{k_{bf}}{k_{nf}}y_{7}-0.5\mathrm{Pr}{\left(1-\varphi \right)}^{2.5}(1-\varphi +\varphi \frac{\rho_{CNT}}{\rho_{bf}})y_{1}y_{7}\\ -\frac{4}{Nr}{\left(1+(\theta_{r}-1)\theta \right)}^{2}\xi (\theta_{r}-1)\theta \prime^{2}\\ -\frac{4}{3Nr}{\left(1+(\theta_{r}-1)\theta \right)}^{3}0.5\theta \prime -\frac{4Ec\mathrm{Pr}}{{\left(1-\varphi \right)}^{2.5}}\xi y_{3}^{2}\\ -2\alpha \beta Ec\mathrm{Pr}{\left(y_{5}-y_{2}\right)}^{2}-2Ec\mathrm{Pr}(\frac{\lambda }{{\left(1-\varphi \right)}^{2.5}}+\frac{M}{1+m^{2})y_{2}^{2}}\\ -0.5\alpha \beta_{T}\mathrm{Pr}(1-\varphi +\varphi \frac{{\left(C_{p}\right)}_{CNT}}{{\left(C_{p}\right)}_{bf}})(y_{8}-y_{6}) \end{array}\right]$$

(34) $$\begin{array}{l} \theta_{p}=y_{8},\\ \theta_{p}\prime =yy_{4},\\ yy_{4}=\frac{\gamma \beta_{T}(1-\varphi +\varphi \frac{{\left(C_{p}\right)}_{CNT}}{{\left(C_{p}\right)}_{bf}})}{y_{4}}(y_{8}-y_{6}). \end{array}$$

With boundary conditions

(35) $$\begin{array}{l} y_{1}(a)=\frac{a}{2}\varepsilon ,y_{2}(a)=\frac{\varepsilon }{2},y_{4}(a)=\frac{a}{2}\varepsilon ,y_{5}(a)=\frac{\varepsilon }{2},\\ y_{6}(a)=1,y_{2}(\infty )=0,y_{6}(\infty )=0,y_{8}(\infty )=0. \end{array}$$

Table 3 depicts the validation of the obtained results by comparing with already published articles in limiting case. This endorses the truthfulness of the presented mathematical model. 

Table 4 illustrates the numerically calculated values of the skin friction coefficient for numerous estimates of α, $\phi_{d},$ $F_{r},$ β, M, and m. It is noticed that value of the drag force coefficient is higher for α, $\phi_{d},$ $F_{r},$ and M, but it declines for estimates of β and m for both types of CNTs. Likewise, Table 5 portrays the Nusselt number for $F_{r}$, α, β, m, $N_{r},$ and $\theta_{r}$. It is comprehended that heat transfer rate is higher in case of α and $F_{r},$ but converse behavior is seen for β, m, $N_{r}$, and $\theta_{r}$ for SWCNTs and MWCNTs. 

## 7. Results and Discussion

**Figure 2:**
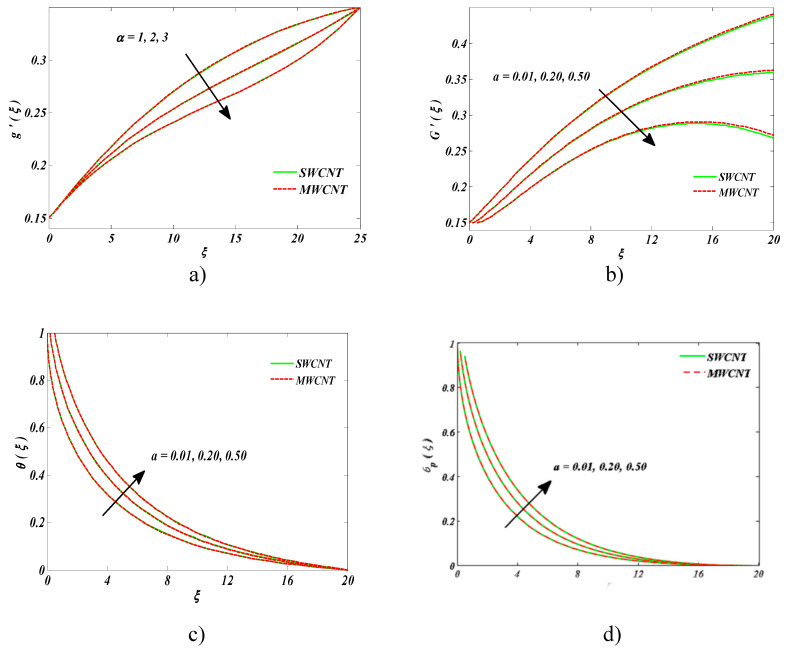
Impact of a on (**a**) nanofluid velocity, (**b**) the velocity of the dust phase, (**c**) nanofluid temperature, and (**d**) temperature of the dust phase.

**Figure 3:**
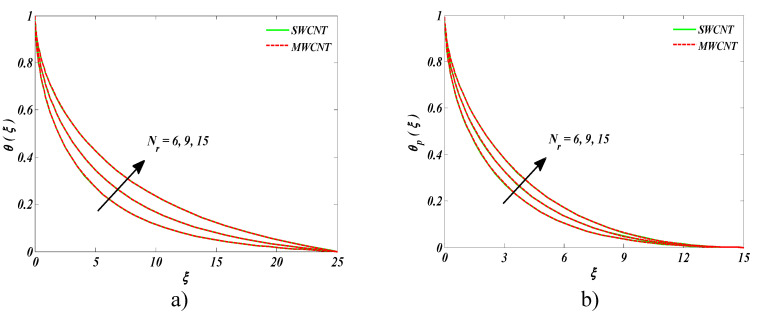
Impact of $N_{r}$ on (**a**) nanofluid temperature and (**b**) temperature of the dust phase.

**Figure 4:**
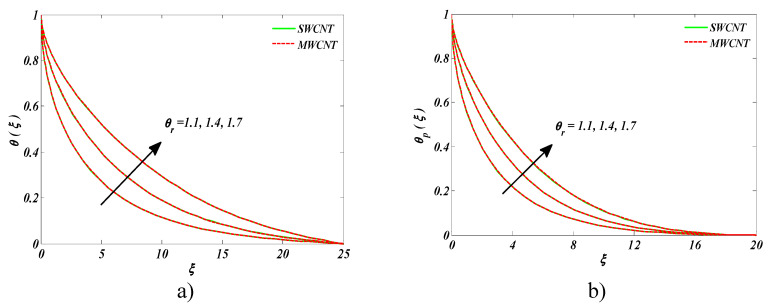
Impact of $\theta_{r}$ on (**a**) nanofluid temperature and (**b**) temperature of the dust phase.

**Figure 5:**
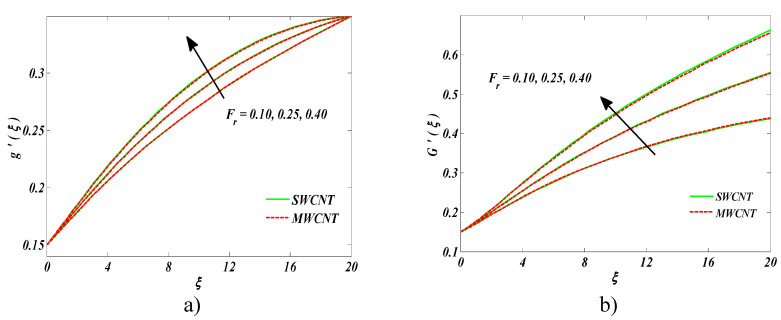
Impact of $F_{r}$ on (**a**) nanofluid velocity and (**b**) the velocity of the dust phase.

**Figure 6:**
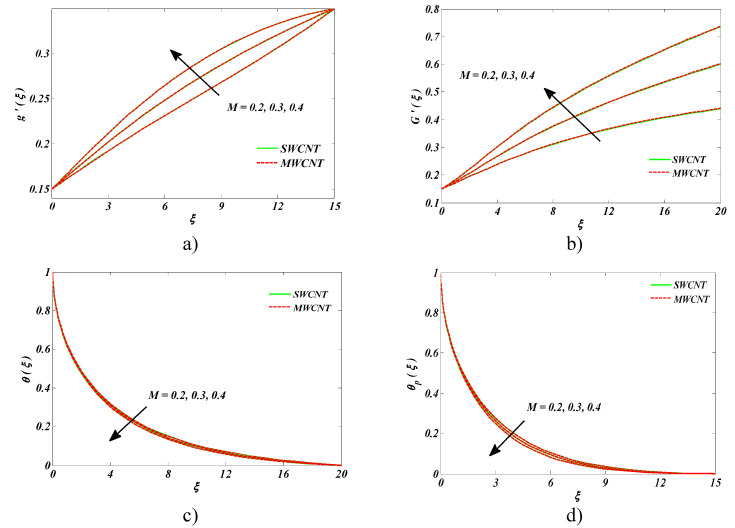
Impact of M on (**a**) nanofluid velocity, (**b**) the velocity of the dust phase, (**c**) nanofluid temperature, and (**d**) temperature of the dust phase.

**Figure 7:**
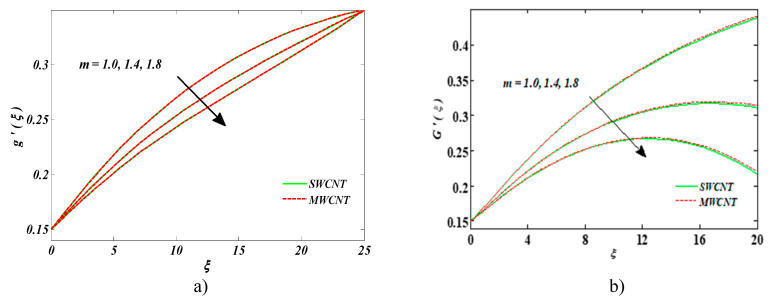
Impact of m on (**a**) nanofluid velocity and (**b**) the velocity of the dust phase.

**Figure 8:**
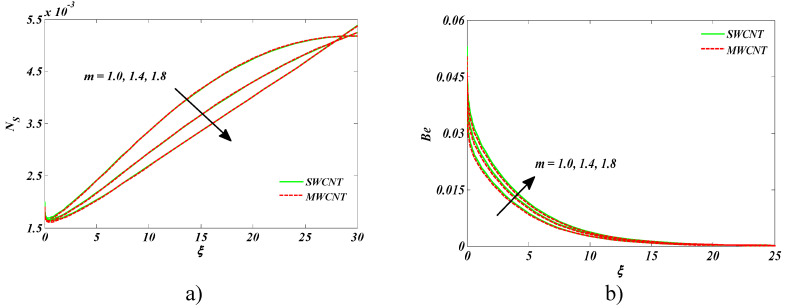
Impact of m on (**a**) entropy generation number and (**b**) Bejan number.

**Figure 9:**
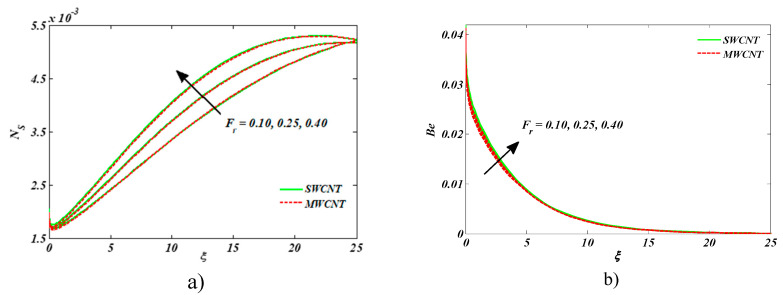
Impact of $F_{r}$ on (**a**) entropy generation number and (**b**) Bejan number.

**Figure 10:**
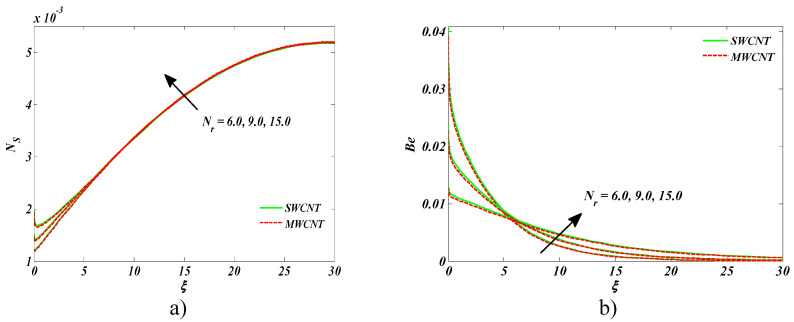
Impact of $N_{r}$ on (**a**) entropy generation number and (**b**) Bejan number.

This segment was devoted to envisioning the physical insight for graphical illustration Figure 2, Figure 3, Figure 4, Figure 5, Figure 6, Figure 7, Figure 8, Figure 9 and Figure 10. We took the fixed values of the parameters throughout the study as (*α* = 0.01, *ε* = 0.3, *ϕ* = 0.04, *ϕ_d_* = *F_r_* = *λ* = *Ec* = 0.1, *α* = *m* = *γ* = 1, *β* = *β_T_* = 0.5, *M* = 0.2, *N_r_* = 6, *θ_r_* = 1.1,) and Pr=6.8. Figure 2a,b exemplify the impacts of needle’s size “a” on the nanofluid velocity and velocity of the dust phase, respectively. It was comprehended that velocities were declining functions of the needle size in the case of CNTs of both types. Physically speaking, both velocities were highly dependent on the size of the needle. Increasing the needle’s size lowered the velocities, which was obvious. An opposite trend was witnessed in the case of Figure 2c,d. It was witnessed that temperature was dominant in the case of SWCNTs as compared to MWCNTs. This was because MWCNTs have lower thermal conductivity than SWCNTs. 

The impact of radiation parameter *Nr* on the nanofluid temperature and temperature of the dust phase could be seen in Figure 3a,b. Owing to higher radiation, more heat was transmitted to both nanofluid and the dust phase. Eventually, the augmented temperature in both cases, i.e., nanofluid and dust fluid, was witnessed. 

Variation in temperature ratio parameter $\theta_{r\;}$ on temperatures of the nanofluid and dust fluid is depicted in Figure 4a,b, respectively. $\theta_{r\;}$ is the quotient of the wall temperature to ambient temperature. Larger values of $\theta_{r\;}$ meant sturdier wall temperature than the ambient temperature. Higher estimates of $\theta_{r\;}$ resulted in a rise in the temperature of both fluids in the case of both CNTs. 

Figure 5a,b revealed the nanofluid velocity and dust phase for the Darcy–Forchheimer parameter *Fr*. It is learned that both velocity functions are diminishing for the growing values of *Fr* [29]. Actually, higher estimates of *Fr* produced resistance in nanofluid motion for both CNTs that ultimately dropped nanofluid and dust fluid velocities. 

The upshot of magnetic parameter *M* on associated distributions is described in Figure 6a–d. Upon increasing the number of dust particles into the fluid, the drag force was strengthened, and more resistance to the fluid flow was experienced, and, eventually, a decrease in both velocities was witnessed. An opposing trend was identified for the temperature field and the dust fluid temperature, which was obviously owing to sturdier *M*. 

Figure 7a,b are outlined to perceive the impact of Hall current parameter *m* on both velocities. It was detected that velocities were mounting functions of *m.* Larger estimates of *m* enforced the damping force, and, eventually, velocities were strengthened. 

The impression of Hall current *m* on entropy generation and the Bejan number is shown in Figure 8a,b. Larger values of *m* lowered the temperature that resulted in a drop of entropy generation as well. An inverse behavior was seen for the Bejan number against *m.*

Figure 9a,b is plotted for entropy generation and Bejan number for Forchheimer parameter *Fr.* An upsurge was visualized in both cases. Larger estimates of the inertial coefficient boosted the disorderliness and caused it to intensify *Ns* and *Be*. 

Figure 10a,b is sketched to comprehend the upshot of radiation parameter *Nr* on entropy generation and the Bejan number, respectively. An enhancement in both entropy generation and the Bejan number was witnessed versus the radiation parameter. This was all because of the heightened energy systems, owing to larger estimates of Nr. 

## 8. Final Remarks

In the present exploration, Hall current sequel on the Darcy–Forchheimer H_2_O-CNTs dusty nanofluid solution over a thin needle was investigated numerically. The novelty impacts of nonlinear thermal radiation with other effects were accompanied by entropy analysis. The leading outcomes of the investigation are appended as follows:

⮚ Bejan number increased for larger values of Darcy–Forchheimer number.
⮚ Velocity was on the decline once the size of the needle and Darcy–Forchheimer parameter’s values were enhanced.
⮚ Higher estimates of Hall current parameter escalated the velocity profiles for both CNTs.
⮚ An upsurge in entropy generation and the Bejan number was witnessed versus the radiation parameter.
⮚ Sturdier magnetic field diminished the velocity of the fluid.
⮚ Skin friction coefficient declined for growing estimates of dust particles’ mass concentration.

## Figures and Tables

**Table 1:** Properties of the nanofluid defined for the presented model [46].

Properties	Nano-Fluid
Density	$\rho_{nf}=\left(1-\phi \right)\rho_{bf}+\phi \rho_{CNT}$
Heat capacity	${\left(\rho C_{p}\right)}_{nf}=\left(1-\phi \right){\left(\rho C_{p}\right)}_{bf}+\phi {\left(\rho C_{p}\right)}_{CNT}$
Viscosity	$\mu_{nf}=\frac{\mu_{bf}}{{\left(1-\phi \right)}^{2.5}}$
Thermal conductivity	$\frac{k_{nf}}{k_{bf}}=\frac{\left(1-\phi \right)+2\phi \left(\frac{k_{CNT}}{k_{CNT}-k_{bf}}\right)ln\left(\frac{k_{CNT}+k_{bf}}{2k_{bf}}\right)}{\left(1-\phi \right)+2\phi \left(\frac{k_{bf}}{k_{CNT}-k_{bf}}\right)ln\left(\frac{k_{CNT}+k_{bf}}{2k_{bf}}\right)}$

**Table 2:** Thermo-physical features of the base fluid H_2_O and CNTs [46].

Thermo-Physical Properties	H_2_O	SWCNT	MWCNT
***C_p_* (*j/kg*)*K***	4179	425	796
***ρ* (*kg*/*m*^3^)**	997.1	2600	1600
***k* (*W/mK*)**	0.613	6600	3000
***Prandtl number* (*Pr*)**	6.8	−	−

**Table 3:** Validation of the existing model for the values of  g ″(a) when $\varepsilon =\phi_{d}=\lambda =F_{r}=\alpha =\beta =M=m=0.$

a	Ishak et al. [47]	Chen and Smith [42]	M. Idrees Afridi et al. [43]	Present Results
0.1	1.2888	1.28881	1.28881	1.28508
0.01	8.4924	8.49244	8.49233	8.4878
0.001	62.1637	62.16372	62.16370	62.1594

**Table 4:** Skin friction coefficient against different parameters.

a	$\phi_{d}$	$F_{r}$	β	M	m	Skin Friction Coefficient	
						SWCNT	MWCNT
0.001						0.00184647	0.00184157
0.01						0.00583012	0.00581468
0.2						0.02591430	0.02584880
	0.1					0.00583012	0.00581468
	2.0					0.00583276	0.00581722
	3.5					0.00583491	0.00581927
		0.10				0.00583012	0.00581468
		0.25				0.00644651	0.00640784
		0.4				0.00706302	0.00700110
			1.0			0.00583012	0.00581468
			2.0			0.00582586	0.00581043
			3.0			0.00582165	0.00580623
				0.2		0.00583012	0.00581468
				0.3		0.00711701	0.00710157
				0.4		0.00840431	0.00838888
					1.0	0.00583012	0.00581468
					1.4	0.00499559	0.00498015
					1.8	0.00447082	0.00445537

**Table 5:** Numerical values of Nusselt number against different parameters.

$F_{r}\;$	α	β	m	$N_{r}$	$\theta_{r}$	Nusselt Number	
						SWCNT	MWCNT
0.10						1.10739	1.05489
0.25						1.12048	1.06695
0.40						1.13570	1.08087
	1.0					1.10739	1.05489
	2.0					1.28855	1.22836
	3.0					1.44647	1.37965
		0.3				1.12089	1.06788
		0.5				1.10739	1.05489
		0.9				1.09958	1.04738
			1.0			1.10739	1.05489
			1.4			1.09857	1.04640
			1.8			1.09329	1.04131
				6.0		1.10739	1.05489
				9.0		0.80951	0.77264
				15.0		0.59158	0.56589
					1.1	1.10739	1.05489
					1.4	0.80635	0.77013
					1.7	0.71356	0.68292

## Nomenclature

r	Coordinate measured in radial direction
(u,v)	Velocity components along x and r directions
$\mu_{nf}$	Effective dynamic viscosity of nanofluid
$\rho_{nf}$	Density of nanofluid
$\upsilon_{nf}$	Kinematic viscosity of nanofluid
$k^{*}$	Darcy-permeability of the porous medium
$C_{b}$	Drag coefficient
$\phi_{d}$	Volume fraction of dust particles
K	Stokes resistance
N	Number density of dust particles
σ	Electric conductivity
$B_{0}$	Applied magnetic field
m	Hall parameter
*m_d_*	Mass concentration of the dust particles
*k_nf_*	Effective thermal conductivity of the nanofluid
(*ρC_p_*)*_nf_*	Effective heat capacitance of the nanofluid
*N*_1_	Density of the particle phase
*τ_v_*	Relaxation time of dust particles
*τ_T_*	Thermal equilibrium time
*τ_w_*	Shear stress at the surface
${\dot{S}}^{\prime \prime \prime }{}_{GEN}$	Entropy generation rate per unit volume
$\left(u_{p},v_{p}\right)$	Velocity components of particle phase in *x* and *r* directions
$c_{m}$	Specific heat of the dust particles
$u_{w}$	Velocity of the moving needle
$u_{\infty }$	Velocity outside the boundary layer
T	Dimensional temperature of the nanofluid
$T_{p}$	Temperature of the dust particle
$T_{w}$	Constant surface temperature of the thin needle
$T_{\infty }$	Ambient temperature
λ	Porosity parameter
$F_{r}$	Forchheimer parameter
α	Dust particles mass concentration
β	Fluid particle interaction parameter for velocity
M	Magnetic field parameter
Pr	Prandtl number
$N_{r}$	Nonlinear radiation parameter
$\theta_{r}$	Temperature ratio parameter
Ec	Eckert number
$\beta_{T}$	Fluid particle interaction parameter for temperature
γ	Ratio of specific heat
$q_{w}$	Surface heat flux
$N_{S}$	Entropy generation number
